# Combined hepatocellular-cholangiocarcinoma: A rare and challenging diagnostic entity – A Case Report

**DOI:** 10.18632/oncoscience.658

**Published:** 2026-05-19

**Authors:** Faten Limaiem, Zied Hadrich, Aziz Atallah, Mohamed Hajri, Sahir Omrani

**Affiliations:** ^1^Department of Pathology, Hospital Mongi Slim La Marsa, Marsa, Tunisia; ^2^Department of Surgery, Hospital Mongi Slim La Marsa, Marsa, Tunisia

**Keywords:** liver cancer, combined hepatocellular-cholangiocarcinoma, pathology, immunohistochemistry, case report

## Abstract

Background: Combined hepatocellular-cholangiocarcinoma (cHCC-CCA) is a rare and aggressive liver cancer, representing 2–5% of primary hepatic malignancies. Its mixed hepatocytic and cholangiocytic features pose major diagnostic challenges, and the absence of standardized treatment guidelines limits effective management.

Case Presentation: A 61-yearold man with type 2 diabetes, hypertension, and chronic hepatitis B presented with right hypochondrial pain. Laboratory tests revealed elevated alpha-fetoprotein (247 ng/mL). Imaging showed a heterogeneous mass in liver segment V with capsular retraction, hilar lymphadenopathy, and atypical enhancement. Histological examination of the CT-guided liver biopsy suggested hepatocellular carcinoma. However, this proved to be a key diagnostic pitfall, as the complete resected specimen revealed a mixed tumor: final diagnosis of combined hepatocellularcholangiocarcinoma (cHCC-CCA) with approximately 20% hepatocellular and 80% cholangiocarcinoma components.

Clinical Discussion: This case underscores the limitations of preoperative biopsy in diagnosing cHCC-CCA, highlighting the risk of misdiagnosis. Radiological features often reflect the predominant histological component but can be nonspecific due to tumor heterogeneity. Definitive diagnosis requires thorough histopathological examination with immunohistochemistry.

Conclusions: cHCC-CCA poses a significant diagnostic and therapeutic challenge. Recognizing this mixed phenotype in complex liver masses is crucial. Histopathological confirmation and complete surgical resection remain key to optimal outcomes. Detailed case reports are essential to improve diagnostic criteria, elucidate molecular biology, and guide evidence-based management for this rare malignancy.

## INTRODUCTION

Combined hepatocellular-cholangiocarcinoma (cHCC-CCA) is a rare, aggressive primary liver malignancy characterized by the dual hepatobiliary differentiation within a single tumor [1]. Representing approximately 2–5% of primary hepatic cancers, it exhibits distinct biological and pathological complexity that sets it apart from conventional hepatocellular carcinoma (HCC) and intrahepatic cholangiocarcinoma (iCCA) [1–3]. Clinically, cHCC-CCA follows a more aggressive course and carries a poorer prognosis than HCC [3]. Its pronounced morphological and phenotypic heterogeneity often leads to diagnostic uncertainty and misclassification [1, 2]. Although advances in imaging and histopathology have improved early detection, significant gaps persist regarding its pathogenesis, prognostic factors, and therapeutic management [1, 2]. The tumor predominantly affects men with underlying cirrhosis or chronic liver disease, yet standardized treatment protocols are lacking due to its rarity and biological diversity. Consequently, large-scale studies remain limited, and treatment response and long-term outcomes are not well defined [1, 2]. This report highlights the diagnostic challenges of cHCCCCA through detailed clinicopathological, radiological, and immunohistochemical correlation. It emphasizes the limitations of preoperative biopsy in heterogeneous liver tumors and the importance of multidisciplinary evaluation for accurate diagnosis and optimal management. This case report has been reported in line with the SCARE checklist [4].

## CASE PRESENTATION

### Patient history and presenting complaint

A 61-year-old male with a medical history of type 2 diabetes, arterial hypertension, and chronic hepatitis B, managed with Entecavir (1 tablet/day), presented with a six-month history of persistent right hypochondrial pain.

### Physical examination findings

On admission, the patient was alert, fully oriented, and in no acute distress. He was hemodynamically stable, with a normal respiratory rate and afebrile. Abdominal examination showed no tenderness, palpable masses, hepatomegaly, or splenomegaly. There were no signs of chronic liver disease, hepatocellular insufficiency, or portal hypertension. Additionally, no peripheral edema or ascites were observed.

### Diagnostic workup

Laboratory investigations showed hemoglobin 13.6 g/dL, platelets 172,000/μL, prothrombin time 93%, serum albumin 43.5 g/L, and elevated alpha-fetoprotein (AFP) at 247 ng/mL (reference <24 ng/mL). Carbohydrate antigen 19-9 (CA 19-9) was not assessed because the initial clinical and radiological findings favored HCC. Liver and renal function tests, as well as electrolytes, were within normal limits, with no evidence of jaundice or cholestasis (normal total bilirubin, AST, ALT, ALP, and GGT). Viral serology confirmed chronic hepatitis B infection. Abdominal ultrasound revealed a hypoechoic, non-vascularized hepatic nodule. Contrast-enhanced CT scan (Figures 1 and 2) demonstrated a heterogeneous, spontaneously hypodense mass in segment V, adjacent to the gallbladder bed, with mild contrast enhancement. The lesion measured 58 × 44 × 69 mm, was associated with capsular retraction, and accompanied by hilar lymphadenopathy, the largest measuring 21 × 11 mm. Abdominal MRI confirmed a lobulated mass in segment V, showing intermediate T2 signal intensity with necrotic areas, progressive peripheral enhancement, absence of arterial wash-in and venous wash-out, and diffusion hyperintensity. Histological examination of the CT-guided liver biopsy, combined with immunohistochemical analysis, confirmed the diagnosis of hepatocellular carcinoma.

**Figure 1:**
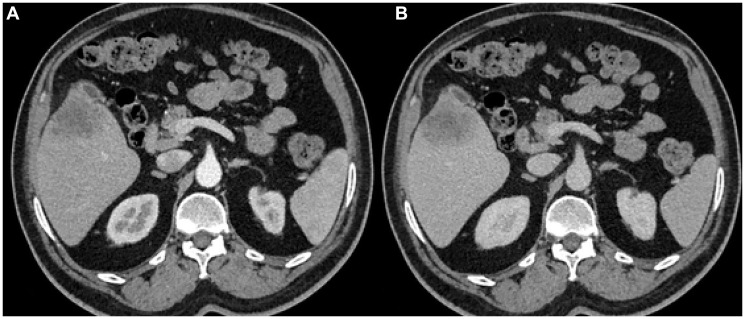
Axial abdominal CT scan in arterial phase (**A**) and portal venous phase (**B**) showing a hypodense, heterogeneous lesion in segment V of the liver. The lesion demonstrates no arterial phase enhancement (wash-in) or portal venous phase washout.

**Figure 2:**
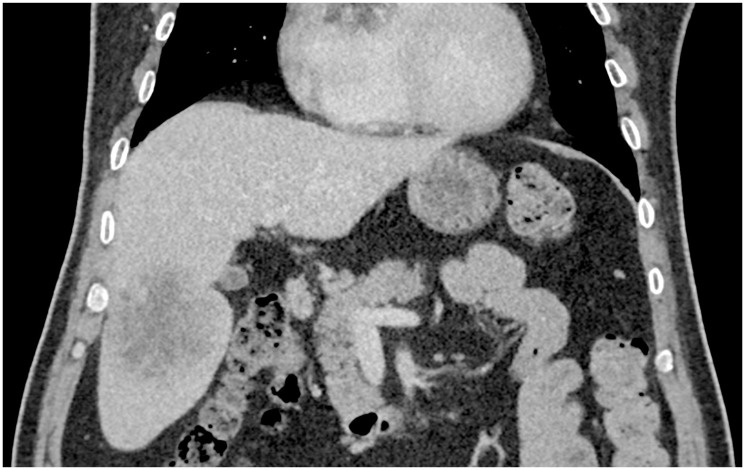
Coronal abdominal CT scan in the portal venous phase showing a hypodense lesion in segment V of the liver.

### Treatment and follow-up

The patient underwent resection of segment V. The postoperative course was uneventful, and the patient was discharged on postoperative day 4. He is currently being closely monitored through regular follow-up.

### Pathology findings

The surgical specimen weighed 302 g and measured 14 × 7.5 × 7 cm. Gross examination revealed a well-circumscribed subcapsular tumor. On cut section, the tumor was whitish-yellow with foci of necrosis and fibrosis, measuring 7 × 6.5 × 6.3 cm (Figure 3). Histologically, the hepatic mass was consistent with a malignant epithelial tumor displaying a dual component. The hepatocellular carcinoma component accounted for approximately 20% of the tumor and was poorly differentiated (Figure 4A, 4B), while the cholangiocarcinoma component represented about 80% and ranged from well- to moderately differentiated (Figure 4C, 4D). Immunohistochemical analysis showed that the tumor cells were negative for CDX2, CK20, PSA, and TTF1. PSA (prostate-specific antigen) immunohistochemistry was included in the diagnostic panel to exclude metastatic prostate adenocarcinoma, given the patient’s age and sex, as the liver is a common site for metastasis from prostatic primary tumors. The negative PSA staining effectively ruled out this possibility, thereby supporting the diagnosis of primary liver malignancy. The hepatocellular component was positive for Glypican-3, HepPar1 (Figure 5A, 5B), and AFP, whereas the cholangiocarcinoma component demonstrated strong positivity for CK19 and CK7 (Figure 5C, 5D). The final pathological diagnosis was cHCCCCA. The resection margins were histologically free of tumor (R0), and the examination documented the absence of microvascular, lymphatic, and perineural invasion. All examined regional lymph nodes were free of metastasis.

**Figure 3:**
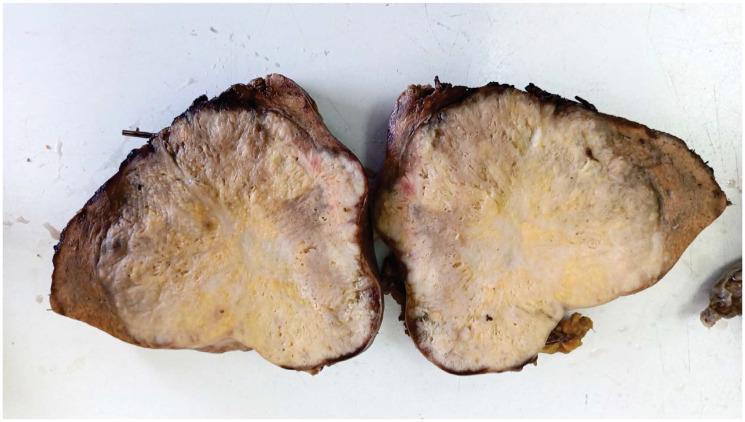
Macroscopic findings: Segment V hepatectomy specimen showing a well-circumscribed subcapsular tumor. On cut section. The tumor appears whitish-yellow with areas of necrosis and fibrosis, measuring 7 × 6.5 cm.

**Figure 4:**
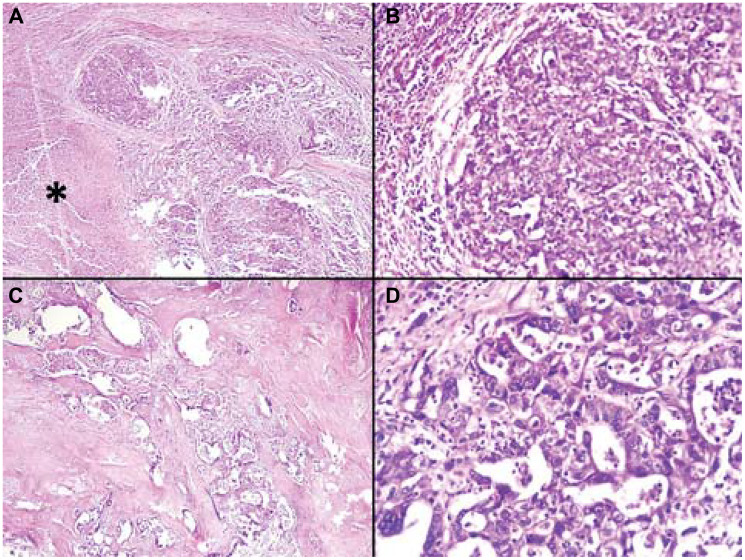
(**A**) Histological section demonstrating a poorly differentiated hepatocellular carcinoma component, characterized by tumor cells arranged in solid sheets, (Hematoxylin and eosin, × 100). (**B**) Histological section showing a poorly differentiated component of hepatocellular carcinoma, characterized by tumor cells arranged in solid sheets, displaying moderate nuclear atypia, (Hematoxylin and eosin, × 200). (**C**) Histopathological section showing a focus of cholangiocarcinoma, characterized by glandular structures of variable size lined by atypical malignant epithelial cells, surrounded by a prominent desmoplastic stroma, (Hematoxylin and eosin, × 100). (**D**) Histopathological section demonstrating a component of cholangiocarcinoma, characterized by closely apposed glandular structures composed of malignant epithelial cells embedded in a dense desmoplastic stroma, (Hematoxylin and eosin, × 400).

**Figure 5:**
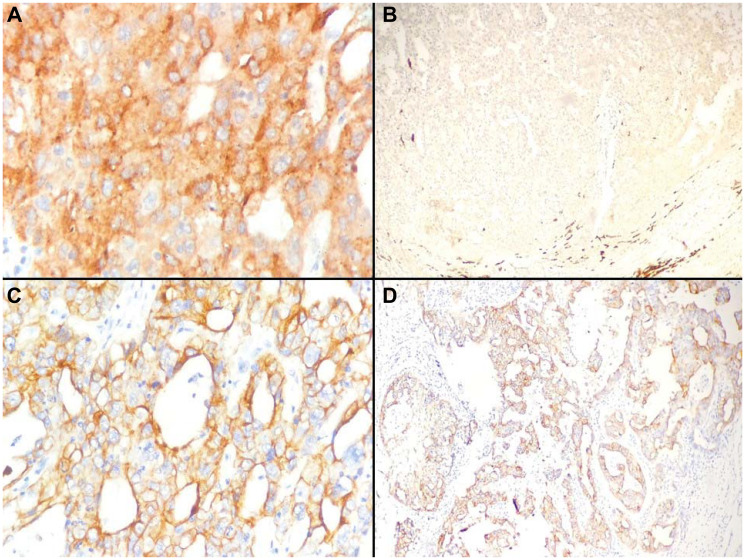
(**A**) Immunohistochemical staining demonstrates Glypican 3 positivity in the tumor cells, (Immunohistochemistry, × 400). (**B**) The tumor cells exhibit moderate HepPar1 expression on immunostaining, (Immunohistochemistry, × 200). (**C**) Tumor cells show positive immunoreactivity for CK19, (Immunohistochemistry, magnification × 400). (**D**) Positive immunostaining of tumor cells with CK 7, (Immunohistochemistry, × 200).

## DISCUSSION

cHCC-CCA is a rare and heterogeneous primary liver malignancy characterized by the coexistence of hepatocytic and cholangiocytic differentiation within the same tumor. It predominantly affects middle-aged or elderly men with a background of chronic liver disease, most often hepatitis B or C infection, alcohol-related injury, or metabolic dysfunction [1–3, 5]. Our patient, a 61-year-old man with chronic hepatitis B, fits this classical profile. The same predisposing context was observed in the cases described by Chen et al. and Deiana et al., both of whom reported underlying viral hepatitis and cirrhosis [2, 6]. Clinically, cHCC-CCA presents with nonspecific manifestations such as abdominal discomfort, fatigue, or jaundice, which frequently delay diagnosis [2, 6]. Laboratory findings are variable and often nondiagnostic.

Alpha-fetoprotein (AFP) levels tend to rise in hepatocellular-predominant tumors, whereas CA 19-9 increases in cholangiocytic forms; concurrent elevation of both is observed in only a minority of patients [2, 7]. In our case, AFP was markedly elevated, while CA 19-9 was not assessed because the initial clinical and radiological findings favored HCC. The radiological appearance of cHCC-CCA is variable, as it directly reflects the tumor’s predominant histological component. This correlation is fundamental to preoperative assessment. When the hepatocellular element is dominant, imaging typically mirrors conventional HCC, characterized by arterial phase hyperenhancement followed by contrast “washout” in later phases [8, 9]. Conversely, when CCA predominates, as in the present case, the features align with those of iCCA. These characteristic findings include peripheral rim enhancement in the arterial phase, progressive and often incomplete centripetal filling in the delayed phases, and associated features such as capsular retraction and lobulated margins [8, 9]. In our patient, the contrast-enhanced studies revealed a heterogeneous mass in segment V demonstrating progressive peripheral enhancement without arterial wash-in, accompanied by capsular retraction and hilar lymphadenopathy. This constellation of findings strongly suggested a CCA-predominant phenotype. The subsequent pathological analysis provided a striking confirmation, revealing that 80% of the tumor consisted of CCA. Histopathological examination remains the gold standard for diagnosis. According to the 2019 WHO classification, cHCC-CCA must demonstrate unequivocal hepatocytic and cholangiocytic morphologies within the same tumor, excluding collision lesions [5, 10]. Immunohistochemistry supports lineage confirmation.

HepPar-1, Arginase-1, and Glypican-3 highlight hepatocytic differentiation, whereas CK7 and CK19 mark biliary epithelium [5, 10]. In our case, the preoperative biopsy revealed only hepatocellular features, resulting in an initial misdiagnosis of HCC. The resection specimen, however, displayed dual morphology with approximately 20% hepatocellular and 80% cholangiocellular differentiation. This discordance between the biopsy and final resection histology represents the central diagnostic pitfall in managing cHCC-CCA. It powerfully illustrates the critical limitations of needle biopsy in accurately sampling highly heterogeneous tumors, a challenge similarly emphasized by Chen et al. and Deiana et al., whose preoperative samples also captured only one component of the mixed tumor [2, 6]. The main differential diagnoses of cHCC-CCA are HCC, CCA, reactive bile ductules, and poorly differentiated carcinomas. HCC shows pseudoglands, HepPar-1/arginase-1/glypican-3 positivity, and no desmoplasia. CCA produces mucin, expresses CK7/CK19, and may trap hepatocytes [5, 10]. Reactive ductules are inflammatory, non-mass-forming, and non-desmoplastic. Poorly differentiated carcinomas overlap features, requiring immunohistochemistry.

Recent molecular studies have shown that the hepatocellular and cholangiocarcinoma components frequently share key mutations, including TP53, TERT-promoter, and AXIN1, supporting a monoclonal origin from hepatic progenitor cells capable of bidirectional differentiation. This shared genetic background, combined with divergent phenotypic differentiation, contributes to tumor heterogeneity and partly explains the diagnostic challenges and variable therapeutic responses observed in cHCC-CCA. It also highlights the potential role of molecular profiling in identifying targets for personalized therapy [11, 12]. There are no established standard therapies for cHCC-CCA, with surgical resection remaining the only potentially curative option, though feasible in a minority of patients. According to National Comprehensive Cancer Network guidelines, surgery is recommended for patients with well-compensated liver function (Child-Pugh class A) or selected class B patients, provided tumor location is favorable, future liver remnant is sufficient, and portal hypertension is absent. In advanced-stage disease, resection may be reconsidered following conversion therapy [13, 14]. Non-surgical approaches are largely adapted from HCC and intrahepatic CCA. Transarterial chemoembolization (TACE) shows limited efficacy with Mukund et al. reporting significantly worse outcomes for cHCC-CCA compared with HCC disease-free survival 1.5 vs. 7.5 months, overall survival 12 vs. 28 months, and objective response rate 25% vs. 91% findings [14, 15]. Tumor vascularity is critical: response rates reach 85% in hypervascular tumors versus 10% in hypovascular lesions. Systemic chemotherapy, most commonly gemcitabine combined with platinum agents, may provide modest benefit in advanced disease; however, optimal treatment selection remains uncertain given the absence of standardized therapeutic guidelines for cHCCCCA. [13, 14]. Recent genomic studies have identified actionable mutations in up to one-quarter of cases (BRCA2, ERBB2, IDH1, FGFR2, MET), offering new therapeutic perspectives [16]. Moreover, immunotherapy may represent a promising option for immune-inflamed molecular subtypes [16]. Prognosis of cHCCCCA is generally worse than HCC but intermediate between HCC and CCA. cHCC-CCA shows higher rates of metastasis and recurrence. Poor outcomes are associated with large tumors (>5 cm), multiple or satellite nodules, lymph node or vascular invasion, advanced stage, elevated CA 19-9, and margins <2 cm [2]. In our case, the tumor was solitary, measured 7 cm, and showed no vascular or lymphatic invasion with negative margins. All examined regional lymph nodes were free of metastasis.

## CONCLUSIONS

In conclusion, this case underscores the diagnostic complexity of cHCC-CCA and the essential role of thorough histopathological and immunohistochemical evaluation. Complete surgical resection remains the only curative option, highlighting the need for early recognition and multidisciplinary management. Continued molecular and clinical research is crucial to develop standardized diagnostic criteria and targeted therapeutic strategies for this rare malignancy.
